# Strategies for Regulating Reactive Oxygen Species in Carbon Nitride-Based Photocatalysis

**DOI:** 10.3390/molecules30173586

**Published:** 2025-09-02

**Authors:** Qingyun Liu, Xiaoqiang Li, Yuxiao Chen, Xinhuan Zhang, Bailin Gao, Manqiu Ma, Hui Yang, Saisai Yuan, Qitao Zhang

**Affiliations:** 1 School of Environmental and Chemical Engineering, Jiangsu University of Science and Technology, Zhenjiang 212100, China; lqy18896663673@126.com (Q.L.); 18914283837@163.com (Y.C.); 17615268618@163.com (X.Z.); bojue2021@126.com (B.G.); 18118952310@163.com (M.M.); 2 Zhejiang Meisheng New Materials Co., Ltd., Shaoxing 312025, China; lixiaoqiang@meishenggroup.com; 3 College of Environmental Science and Engineering, Yangzhou University, Yangzhou 225127, China; huiyang5211@yzu.edu.cn; 4 International Collaborative Laboratory of 2D Materials for Optoelectronics, Science and Technology of Ministry of Education, Institute of Microscale Optoelectronics, Shenzhen University, Shenzhen 518060, China

**Keywords:** polymeric carbon nitride, reactive oxygen species, photocatalysis, ROS regulation, redox selectivity

## Abstract

Reactive oxygen species (ROS) are increasingly recognized as decisive actors in photocatalytic redox chemistry, dictating both the selectivity and efficiency of target reactions, while most photocatalytic systems generate a mixture of ROS under illumination. Recent studies have revealed that tailoring the generation of specific ROS, rather than maximizing the overall ROS yield, holds the key to unlocking high-performance and application-specific catalysis. In this context, the selective production of specific ROS has emerged as a critical requirement for achieving target-oriented and sustainable photocatalytic transformations. Among the various photocatalytic materials, polymeric carbon nitride (PCN) has garnered considerable attention due to its metal-free composition, visible-light response, tunable structure, and chemical robustness. More importantly, the tunable band structure, surface chemistry, and interfacial environment of PCN collectively make it an excellent scaffold for the controlled generation of specific ROS. In recent years, numerous strategies including molecular doping, defect engineering, heterojunction construction, and co-catalyst integration have been developed to precisely tailor the ROS profile derived from PCN-based systems. This review provides a comprehensive overview of ROS regulation in PCN-based photocatalysis, with a focus on type-specific strategies. By classifying the discussion according to the major ROS types, we highlight the mechanisms of their formation and the design principles that govern their selective generation. In addition, we discuss representative applications in which particular ROS play dominant roles and emphasize the potential of PCN systems in achieving tunable and efficient photocatalytic performance. Finally, we outline key challenges and future directions for developing next-generation ROS-regulated PCN photocatalysts, particularly in the context of reaction selectivity, dynamic behavior, and practical implementation.

## 1. Introduction

Reactive oxygen species (ROS), including superoxide anion radical (•O_2_^−^) [1,2], hydrogen peroxide (H_2_O_2_) [3,4], hydroxyl radical (•OH) [5,6,7,8], and singlet oxygen (^1^O_2_) [9,10,11,12,13], represent a class of highly reactive intermediates with wide-ranging functions in photocatalytic redox chemistry. Their high oxidative or reductive potential underlies a variety of processes, such as pollutant degradation [14,15], water splitting [16,17,18], organic synthesis [19,20,21], and antibacterial disinfection [22,23,24]. The nature and dynamics of ROS dictate not only the reactivity and selectivity of these reactions, but also their energy efficiency and environmental compatibility. However, uncontrolled or mixed ROS generation often leads to non-selective pathways and poor product yields. Therefore, the ability to selectively generate a target ROS under visible light has emerged as a frontier in photocatalysis research. Over the past decade, considerable efforts have been devoted to engineering photocatalysts that steer ROS formation toward desired species. Classical semiconductor systems have demonstrated efficient ROS generation through band-structure-driven charge separation. Heterojunctions and plasmonic composites have been designed to boost ROS productivity via enhanced carrier dynamics and oxygen activation. More recently, metal–organic frameworks (MOFs) [25,26,27], single-atom catalysts [28,29,30], and 2D materials [31,32,33] have offered novel platforms for modulating interfacial charge transfer and ROS specificity, owing to their tunable coordination environments and rich surface chemistry.

Since Fujishima and Honda’s landmark 1972 demonstration of TiO_2_ photoelectrochemical water splitting first revealed that photoexcited electrons and holes can drive redox chemistry, researchers have steadily unraveled the specific reactive oxygen species (ROS) at play [34]. In the 1990s, the advent of spin-trapping EPR enabled the direct observation of •OH and •O_2_^−^ radicals, cementing the role of ROS in photocatalysis [35]. The field turned towards visible light in 2009 when Wang and co-workers [36] introduced graphitic carbon nitride as an efficient photocatalyst; by 2011, DMPO-EPR and benzoquinone scavenging confirmed that photogenerated electrons in PCN reduce O_2_ to •O_2_^−^. In 2021, time-resolved spectroscopic studies and kinetic modeling by Velo-Gala et al. [37] highlighted the critical involvement of hydroperoxyl (HOO•) intermediates during pollutant degradation. Tailored heterojunctions designed in them demonstrated the precise tuning of ^1^O_2_ versus •OH production on carbon nitride, while photoswitchable surface functionalization strategies enabled the on-demand modulation of ROS pathways. This trajectory, from foundational TiO_2_ discoveries to sophisticated PCN engineering, maps a clear evolution toward materials that not only generate ROS under solar irradiation but do so with unprecedented selectivity and control.

Despite these advances, many conventional systems face limitations in terms of visible-light response, structural stability, and scalability. In this context, polymeric carbon nitride (PCN) [3,5,38,39,40,41,42] has attracted increasing attention as a metal-free, visible-light-active photocatalyst capable of controllable ROS generation. Its unique features, including a moderate bandgap (~2.7 eV), strong π-conjugated framework, and chemical robustness, make it particularly well suited for sustainable redox catalysis. More importantly, the physicochemical attributes of PCN, such as its tunable band structure [43], abundant surface lone pairs, and customizable defect states, offer an ideal platform for modulating ROS generation pathways at the molecular level. Recent studies have shown that the precise structural engineering of PCN enables the selective formation of specific ROS, such as favoring ^1^O_2_ via energy transfer channels, or steering electron–proton coupling to yield H_2_O_2_ instead of •OH. These insights have led to the emergence of ROS-oriented design principles in PCN photocatalysis, in which synthesis, surface chemistry, and cocatalyst strategies are tailored toward a given ROS-driven application. Nonetheless, probing ROS dynamics remains a formidable challenge, as their fleeting lifetimes and overlapping reactivity complicate in situ detection and quantitative analysis during catalytic processes.

**Figure 1:**
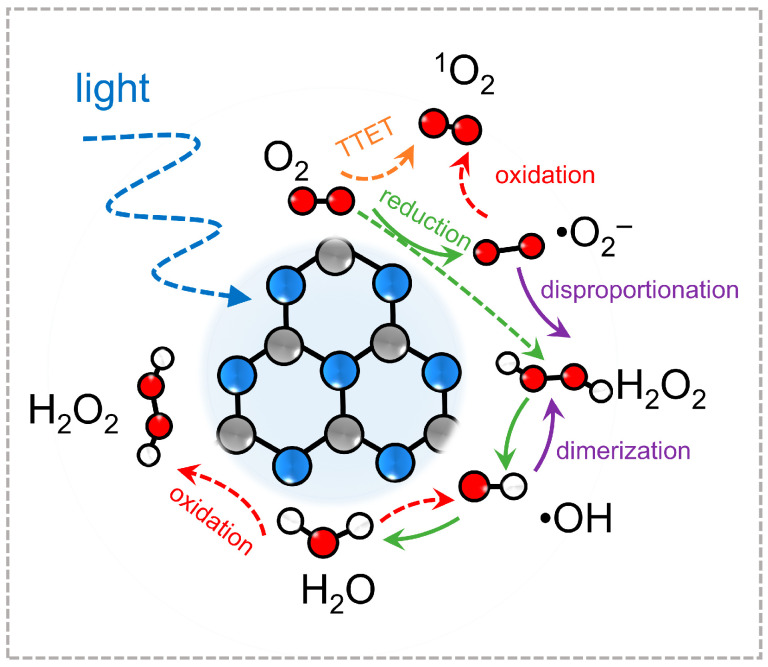
Schematic diagram of the generation of reactive oxygen species on PCN.

To systematically summarize these developments, Figure 1 presents a schematic diagram depicting the landscape of ROS generation and transformation on PCN under light irradiation. Upon excitation, photoinduced electrons and holes initiate multiple oxygen activation pathways. Electrons may sequentially reduce O_2_ to •O_2_^−^, H_2_O_2_, and •OH, while energy transfer routes can yield ^1^O_2_ directly from molecular oxygen. Each ROS pathway involves distinct intermediates and potential interconversions (e.g., the protonation of •O_2_^−^ to •OOH, or the decomposition of H_2_O_2_ to •OH), forming a dynamic and interconnected ROS network on the PCN surface. This diagram highlights both the chemical diversity of ROS available through PCN activation and the need for tailored modulation to harness their full potential in specific reactions. The one-electron reduction of O_2_ to •O_2_^−^ by photogenerated electrons has been experimentally confirmed by p-benzoquinone scavenger tests and DMPO spin-trap EPR measurements of H_2_O_2_. The subsequent dismutation of •O_2_^−^ into H_2_O_2_ is likewise verified via titanium-sulfate colorimetry and iodometric titration. •OH arising from H_2_O_2_ decomposition or Fenton-like reactions at metal sites have been detected by DMPO-EPR, terephthalic acid fluorescence probing, and isopropanol quenching. ^1^O_2_ formation has been confirmed by TEMP-EPR trapping and furfuryl alcohol decay assays. By contrast, direct hole-driven water oxidation to •OH, two-electron O_2_ reduction to H_2_O_2_ on undoped PCN, and certain multi-step ROS interconversions remain at the level of DFT-computed adsorption energies and reaction barriers, with no dedicated radical scavenger or spectroscopic verification to date.

Previous reviews on ROS generation by carbon nitride have largely focused on general strategies such as band-structure engineering, defect modulation, and heterojunction construction to enhance overall ROS productivity. These works often treat ROS collectively, with limited discussion of selective generation, mechanistic pathways, or the interplay among •O_2_^−^, H_2_O_2_, •OH, and ^1^O_2_. In contrast, the present review systematically dissects ROS formation on PCN by species, highlights type-specific regulation strategies, and links these to quantifiable ROS yields. It further explores applications beyond pollutant degradation, including selective organic synthesis, environmental disinfection, and energy-related transformations, emphasizing how controlled ROS formation governs reaction selectivity. By integrating advanced characterization techniques, single-atom catalysis, and predictive AI-driven design, this review provides a mechanistically grounded, application-oriented, and forward-looking perspective that extends and complements the prior literature.

## 2. Generation of ROS on PCN

The photocatalytic behavior of polymeric carbon nitride (PCN) is intimately shaped by its ability to mediate diverse reactive oxygen species (ROS), each featuring distinct redox potentials, lifetimes, and reactivities. Once photoexcited, PCN engages in a series of interfacial electron and energy transfer processes with molecular oxygen or water, giving rise to species ranging from moderately reactive •O_2_^−^ to highly oxidative •OH and electronically excited ^1^O_2_. The generation of each ROS is determined by the alignment between PCN’s electronic band structure and the redox potentials of oxygen-related intermediates, as well as by kinetic factors such as charge carrier separation, surface adsorption affinity, and the local reaction microenvironment. Figure 1 schematically summarizes the interconversion network of ROS on PCN, emphasizing the diversity and complexity of possible reaction routes. A clear understanding of these primary generation mechanisms is essential for developing effective strategies in ROS-specific photocatalytic design. In this section, we outline the formation pathways of the major ROS observed in PCN-based systems. Each subsection focuses on a specific ROS, detailing its thermodynamic formation criteria, primary reaction steps, and the typical reaction conditions under which it emerges.

### 2.1. Superoxide Anion Radical

The •O_2_^−^ is often the primary ROS generated upon the photoexcitation of PCN in oxygenated environments. This species forms via a one-electron reduction of molecular oxygen by photoexcited electrons from the conduction band (CB) of PCN:

O_2_ + e^−^ → •O_2_^−^ (1)

The feasibility of this process is fundamentally dictated by the relative position of the CB edge of PCN, which typically lies at approximately −1.3 V vs. RHE at a pH of 7, which is more negative than the standard redox potential of the O_2_/•O_2_^−^ couple (−0.33 V) [44,45]. This favorable thermodynamic alignment enables spontaneous electron transfer from photoexcited PCN to dissolved oxygen, especially under anaerobic-free, neutral-to-alkaline aqueous conditions. In PCN systems, the generation of •O_2_^−^ is influenced not only by the CB potential but also by the efficiency of charge carrier separation and migration to the surface. The extended π-conjugation and layered structure of PCN facilitate charge delocalization, while defect states and surface terminations may serve as anchoring sites for O_2_ adsorption and activation. Adsorbed O_2_ molecules first accept a single electron from the conduction band of photoexcited PCN, forming •O_2_^−^ on the surface. These •O_2_^−^ species can either directly oxidize substrates or undergo protonation to form hydroperoxyl radicals (•OOH). Subsequent electron/proton transfer or disproportionation reactions convert •OOH into H_2_O_2_, which can further decompose via Fenton-like or photolytic processes to generate •OH. Collectively, this sequence establishes a dynamic ROS network in which O_2_ is stepwise transformed from •O_2_^−^ to •OOH, then to H_2_O_2_, and finally to •OH, enabling multiple oxidative pathways on PCN surfaces.

Figure 1 indicates •O_2_^−^ as a central node within the ROS network, serving as a precursor to several other reactive species through sequential proton-coupled electron transfer or dismutation reactions. Due to its moderate redox activity and relatively long lifetime in aprotic or mildly alkaline environments, •O_2_^−^ plays a crucial role in selective oxidation reactions, including pollutant degradation and organic transformations. A clear understanding of •O_2_^−^ generation pathways thus serves as the foundation for constructing ROS-tailored photocatalytic systems based on PCN, particularly in applications that demand controlled oxidative strength and spatially confined reactivity.

### 2.2. Singlet Oxygen

^1^O_2_, the first excited electronic state of molecular oxygen, is a highly reactive yet non-radical ROS that plays a pivotal role in oxidation reactions with high selectivity toward electron-rich organic substrates. In PCN-based photocatalysis, ^1^O_2_ generation can proceed through two fundamentally distinct pathways: energy transfer from the excited photocatalyst to ground-state triplet oxygen (^3^O_2_) or the charge-mediated oxidation of superoxide intermediates.

The first mechanism relies on a triplet–triplet energy transfer (TTET) process, shown as follows [46,47,48,49]:

S_0_ → S_1_ → ISC → T_1_ → TTET → ^1^O_2_ (2)

It begins with the photoexcitation of PCN from the ground singlet state (S_0_) to the first excited singlet state (S_1_). Through intersystem crossing (ISC), a fraction of the excited-state population undergoes spin conversion to yield a long-lived triplet excited state (T_1_). If the energy level of PCN’s T_1_ state exceeds that of singlet oxygen (^1^O_2_, ~0.98 eV), a TTET process can occur, whereby the triplet energy of PCN is transferred to adsorbed ^3^O_2_, forming reactive ^1^O_2_ via the following:

^3^O_2_ + PCN (T_1_) → ^1^O_2_ + PCN (T_0_) (3)

This TTET pathway is spin-allowed and does not rely on electron–hole separation, making it especially relevant in systems in which charge recombination limits redox-driven ROS generation. Although pristine PCN exhibits relatively inefficient ISC due to weak spin–orbit coupling, recent advancements, such as heteroatom doping (e.g., S and Cl), π-extended units, and donor–acceptor modifications, have been shown to enhance the ISC efficiency and triplet-state population, thereby facilitating ^1^O_2_ formation.

An alternative and often dominant route for ^1^O_2_ production in PCN systems involves the oxidation of •O_2_^−^ via photogenerated holes. This pathway couples electron transfer and hole transfer steps in sequence, starting with the reduction of O_2_ to •O_2_^−^ (as described in Section 2.1), followed by the oxidative conversion of •O_2_^−^ by valence band holes (h^+^):

•O_2_^−^ + h^+^ → ^1^O_2_ (4)

This two-step radical pathway is kinetically feasible due to the moderate oxidation potential required for •O_2_^−^ oxidation and the relatively positive valence band edge of PCN (approximately +1.9 V vs. RHE, pH = 7.0). The local concentration of •O_2_^−^ and its spatial proximity to hole-rich surface regions are critical for this mechanism to proceed efficiently. Transient spectroscopic studies and ROS scavenging experiments have provided supporting evidence for this conversion, particularly under aerobic and mildly acidic reaction conditions.

Regardless of the specific pathway, the formation of ^1^O_2_ on PCN surfaces is typically associated with enhanced molecular oxygen adsorption and surface excitation dynamics. Its non-radical nature, selective reactivity, and relatively short diffusion length make it an attractive species for applications requiring controlled oxidation, such as antibacterial treatment, photodynamic therapy, and the degradation of specific pollutants.

### 2.3. Hydrogen Peroxide

Hydrogen peroxide is one of the most synthetically and biologically relevant ROS, and its controlled generation is of particular interest in environmental remediation, selective oxidation, and biomedical applications. However, the photocatalytic formation of H_2_O_2_ is mechanistically intricate, involving multiple possible pathways with distinct energetic demands, intermediates, and selectivity profiles. Especially in PCN-based photocatalysis, the subtle tuning of reaction pathways significantly impacts product selectivity and ROS crossover. To provide a systematic overview, Table 1 summarizes the four major reaction routes leading to H_2_O_2_ formation on PCN: (I) stepwise oxygen reduction through •O_2_^−^ and •OOH, (II) direct two-electron oxygen reduction, (III) water oxidation, and (IV) hydroxyl radical (•OH) coupling. These routes vary in their radical involvement, selectivity, and feasibility under photocatalytic conditions [42,50,51,52].

Among them, pathway I, a sequential reduction process starting from molecular oxygen, is the most prevalent under ambient aerobic conditions [53]. Upon visible-light excitation, photoinduced electrons on PCN reduce O_2_ to •O_2_^−^, which can be further protonated to form •OOH and eventually disproportionate or be reduced to H_2_O_2_. This pathway benefits from the intrinsic CB potential of PCN, which typically lies near −0.8 V (vs. RHE, pH = 7.0), sufficiently negative for O_2_/•O_2_^−^ (−0.33 V) and •OOH/H_2_O_2_ (0.89 V) conversions. However, this route is often accompanied by uncontrolled ROS crossover and radical leakage, which compromises product selectivity and catalyst stability.

In contrast, pathway II, the direct two-electron reduction of O_2_ to H_2_O_2_, bypasses radical intermediates and is more desirable for selective H_2_O_2_ production [54]. This route demands precise control of proton-coupled electron transfer (PCET) steps and is often facilitated by surface hydrogen-bond networks or single-atom cocatalysts that stabilize OOH-like intermediates. PCN materials featuring carbonyl, cyano, or defect-rich edge structures have shown improved selectivity toward this concerted pathway. Although this mechanism offers higher theoretical selectivity (as highlighted in Table 1), it typically requires optimized electronic coupling and reaction site geometry.

Pathway III, involving the oxidation of water molecules to H_2_O_2_, is thermodynamically uphill and kinetically sluggish [55]. While PCN is not an ideal water oxidation catalyst, introducing oxidative cocatalysts such as Co or Mn oxides, or integrating PCN with type II heterojunctions, can enhance this pathway to a certain extent. Nevertheless, it suffers from low selectivity due to competing O_2_ evolution and poor charge separation.

Pathway IV leverages the recombination of two •OH radicals to form H_2_O_2_, a route that is common in advanced oxidation processes but rare in PCN systems due to the typically low concentration and short lifetime of •OH [55]. Generating •OH on PCN requires either photogenerated holes or the activation of surface water through valence band oxidation. The overall low selectivity and degradation-prone nature of this path limit its practical contribution, as noted in Table 1.

### 2.4. Hydroxyl Radical

•OH is among the most potent and non-selective ROS, capable of oxidizing a wide range of organic compounds at diffusion-controlled rates. In photocatalysis, they serve as key oxidative agents for pollutant degradation, microbial disinfection, and advanced oxidation processes [56]. Within PCN-based photocatalytic systems, •OH generation is typically associated with two distinct pathways: direct water oxidation and hydrogen peroxide activation.

The direct oxidation of water molecules by photogenerated holes on PCN is a classical route to •OH production. This process requires that the valence band (VB) potential of PCN be sufficiently positive to overcome the oxidation potential of H_2_O/•OH (+2.73 V vs. RHE, pH = 7.0). Although pristine PCN possesses a VB edge around +1.4 V, which is inadequate for water oxidation to •OH, several strategies have been employed to enhance this capability. However, this pathway suffers from intrinsic limitations. The generation efficiency of •OH via water oxidation is generally low due to poor hole mobility and the sluggish kinetics of the four-electron water oxidation process. Additionally, excessive •OH production may lead to the non-selective overoxidation of the catalyst surface or desired intermediates, highlighting the need for regulated •OH flux.

An alternative and often more accessible route to •OH involves the decomposition of photocatalytically generated H_2_O_2_. As discussed in the previous section, H_2_O_2_ can accumulate on the surface of PCN through multi-step oxygen reduction or water oxidation. Under visible light or with transition metal sites (such as Fe^2+^), H_2_O_2_ undergoes homolytic cleavage or Fenton-like reactions to yield •OH:

H_2_O_2_ + *hv* → 2•OH (5)

H_2_O_2_ + Fe^2+^ → Fe^3+^ + •OH + OH^−^ (6)

This pathway is often favored in neutral or slightly acidic conditions, and its efficiency depends heavily on the surface coordination environment and the presence of redox-active moieties. In PCN systems, edge nitrogen atoms, surface –NH_2_ groups, or coordinated single atoms can catalyze H_2_O_2_ activation and guide the selective generation of •OH near the interface. Compared to water oxidation, H_2_O_2_ decomposition offers a lower energy barrier and higher yield of •OH, albeit at the cost of introducing an additional intermediate. Moreover, this route provides a feedback loop within ROS dynamics, in which an initially benign species (H_2_O_2_) evolves into a more aggressive oxidant (•OH), enabling stepwise reactivity tuning.

### 2.5. Summary

The generation of various ROS on PCN-based photocatalysts involves a complex interplay of redox potentials, photogenerated carrier dynamics, and surface molecular interactions. •O_2_^−^ is typically formed through single-electron O_2_ reduction on the CB, whereas ^1^O_2_ arises via energy transfer or through the oxidation of •O_2_^−^. H_2_O_2_ can be produced through four distinct routes, involving both oxygen and water as precursors. •OH, on the other hand, emerges from either water oxidation or H_2_O_2_ decomposition. These pathways are often interlinked and competing, necessitating the precise modulation of electronic and interfacial properties to direct specific ROS generation.

**Figure 2:**
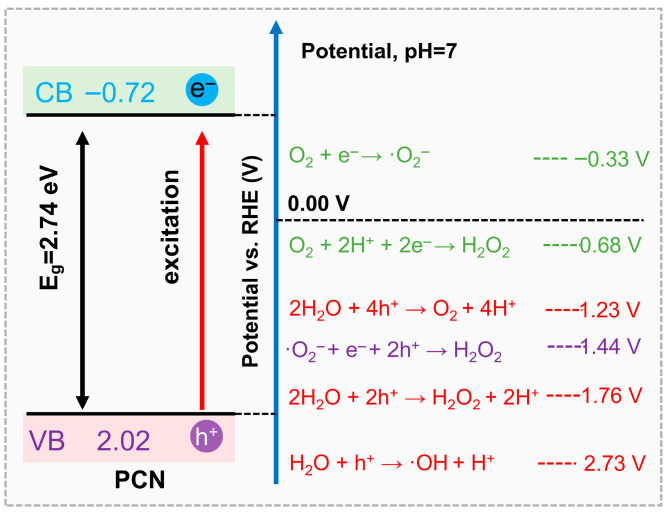
Schematic illustration of the processes involved in PCN-based photocatalytic production of ROS and corresponding energy diagrams.

To visually consolidate these mechanistic insights, Figure 2 presents a schematic overview of the ROS generation routes on PCN, coupled with corresponding redox potentials. The energy diagram clearly illustrates how the positions of the conduction and valence bands of PCN dictate the feasibility of each ROS formation process. This diagram not only summarizes the interconversion of ROS but also highlights the critical thermodynamic thresholds for their formation, offering a unified framework to guide the design of PCN photocatalysts with tailored ROS profiles. It also provides the foundation for the next section, in which we discuss specific strategies to selectively regulate individual ROS production through molecular and electronic engineering.

## 3. Strategies for Regulating Specific ROS in PCN

Building upon the mechanistic understanding of ROS generation on PCN, rational strategies for selective ROS regulation have emerged as a cornerstone for advancing high-efficiency and target-oriented photocatalytic systems. The generation of specific ROS is not solely dictated by thermodynamic redox potentials, but is also intimately governed by the electronic structure, surface chemistry, and local microenvironment of the photocatalyst. In PCN-based systems, in which structural tunability and chemical modularity offer unique advantages, the tailored modulation of reactive pathways allows researchers to steer ROS output towards desired types, concentrations, and spatial distributions. Broadly, strategies for ROS regulation can be categorized into three major approaches: (a) band structure engineering to thermodynamically or kinetically favor particular ROS pathways; (b) defect and surface functionalization to introduce localized charge trapping sites, adsorption centers, or catalytic motifs; and (c) interface construction to promote directional charge transfer and reactive site separation. In the following sections, we systematically discuss the state-of-the-art strategies for regulating each type of ROS in PCN systems, highlighting the structure–activity relationships, typical design motifs, and mechanistic insights that underpin selective ROS modulation.

### 3.1. Energy Band Engineering Toward Selective •O_2_^−^ Formation

•O_2_^−^ occupies a privileged niche among photocatalytic reactive oxygen species: less aggressive than hydroxyl radicals yet sufficiently long-lived to engage in selective oxygenation and downstream, value-adding transformations [57,58,59]. Realizing this control requires more than enhanced light absorption or longer carrier lifetimes, it demands the precise alignment of the catalyst’s electronic states with the thermodynamics of oxygen reduction. In practice, elevating and tailoring the conduction-band edge of PCN, while concurrently engineering interfacial adsorption sites, provides the thermodynamic drive and the local environment needed for efficient one-electron O_2_ reduction without promoting undesirable deeper reduction or radical leakage. In this section, we therefore discuss energy band engineering strategies to favor selective •O_2_^−^ formation on PCN.

**Figure 3:**
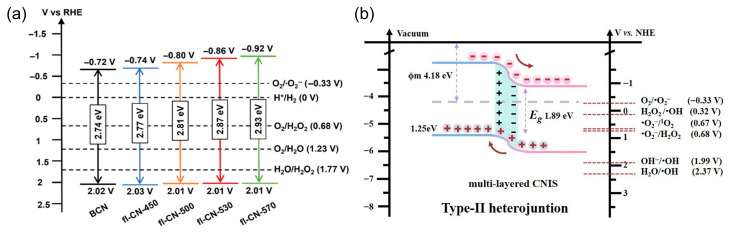
(**a**) Raising CB position to enhance •O_2_^−^ production. Reproduced from ref. [42] with permission from Wiley-VCH. (**b**) The construct of heterojunctions to promote photogenerated charge separation. Reproduced from ref. [14] with permission from Elsevier.

Manipulating the CB edge of PCN to make it more negative increases the thermodynamic driving force for O_2_ reduction. Structural modifications such as heteroatom incorporation [60,61,62,63,64,65,66,67,68] and π-conjugation extension [69,70,71,72] are particularly effective. As shown in Figure 3a, Zong et al. [42] exfoliated bulk PCN into ultrathin few-layer nanosheets via nitrate intercalation and decomposition. This simple hydrothermal approach followed by thermal treatment not only facilitated exfoliation and thickness control down to bi-layers, but also introduced oxygen-containing surface functionalities and modulated the band structure of PCN. The resulting fl-CN exhibited a distinctly elevated conduction band position compared to bulk PCN, thereby significantly increasing the thermodynamic driving force for O_2_ reduction to •O_2_^−^. Meanwhile, the introduction of oxygen species improved surface affinity for O_2_ adsorption, further favoring the formation of superoxide radicals. As a result, the optimized fl-CN-530 photocatalyst achieved a remarkable H_2_O_2_ production rate under visible light, which was 8.8 times that of the pristine bulk PCN.

To enhance the selective generation of •O_2_^−^, promoting efficient photogenerated charge separation is equally critical as elevating the conduction band edge. In typical PCN-based systems, the rapid recombination of photogenerated electrons and holes severely limits the availability of electrons for the reduction of O_2_. Constructing semiconductor heterojunctions with rational band alignment offers an effective strategy to facilitate spatial charge separation, prolong charge carrier lifetimes, and enhance interfacial redox reactions. Specifically, coupling PCN with a secondary semiconductor can create type II or Z-scheme heterostructures that drive the directional migration of photogenerated charges [73,74,75,76,77,78]. For example, in a type II heterojunction, electrons tend to migrate to the semiconductor with a lower conduction band, while holes accumulate in the semiconductor with a higher valence band. In a ZnO/PCN heterojunction system [79], PCN acts as the visible-light-responsive component (band gap ≈ 2.7 eV), while ZnO remains active under UV light. Under visible light, only PCN is excited, generating electrons and holes. Due to the favorable band alignment, the photogenerated electrons on PCN can transfer to the conduction band of ZnO, while holes remain on PCN, leading to efficient charge separation. This spatial separation results in a high density of accumulated electrons on the ZnO surface, where they can efficiently reduce molecular oxygen to generate •O_2_^−^. Meanwhile, the holes retained on PCN can participate in parallel oxidation reactions. This synergistic effect between ZnO and PCN not only improves the lifetime of charge carriers but also enhances the photocatalytic degradation of target pollutants, such as methyl orange, via both •O_2_^−^ and •OH pathways. As shown in Figure 3b, the formation of In-N bonds between CN and In_2_S_3_ establishes a type II heterojunction, shifting the band edges and enriching band-tail states to broaden visible-light absorption and suppress carrier recombination. The ordered sheet-to-sheet architecture with pronounced anisotropy accelerates exciton dissociation and opens multidirectional diffusion channels, enabling rapid electron transfer to surface-adsorbed O_2_ for efficient •O_2_^−^ generation. These synergistic effects collectively underpin the markedly enhanced photocatalytic degradation of tetracycline under visible-light irradiation.

### 3.2. Regulation of Reaction Pathways Toward Selective H_2_O_2_ Formation

H_2_O_2_ occupies a unique position among reactive oxygen species: it is a relatively stable, energy-dense oxidant that can be handled and stored more safely than radical species, and it functions both as a direct oxidant and as a precursor to more potent ROS in situ. The photocatalytic generation of H_2_O_2_ from O_2_ under mild, solar-driven conditions therefore represents a sustainable route to an industrially important chemical, with clear advantages over the traditional anthraquinone process in terms of simplicity, scalability, and environmental impact. Equally important, selective photocatalytic H_2_O_2_ production enables on-demand oxidant supply for water treatment, selective organic synthesis, and decentralized disinfection, while minimizing undesired over-oxidation and radical-mediated degradation. For these reasons, steering photocatalytic reaction pathways to favor H_2_O_2_ formation, rather than non-selective radical generation or full four-electron reduction to H_2_O, is a central objective in the rational design of PCN-based photocatalysts.

The pathway of oxygen reduction in PCN-based photocatalysis, whether proceeding via a direct two-electron reduction to H_2_O_2_ or a stepwise single-electron route involving •O_2_^−^ and •OOH intermediates, is fundamentally governed by the adsorption configuration of O_2_ molecules and the position of the CB. In the direct ORR pathway, molecular oxygen preferentially adopts an end-on adsorption mode, in which only one oxygen atom interacts with the catalyst surface [80]. This configuration favors a concerted 2e^−^ transfer, bypassing radical intermediates and leading to efficient H_2_O_2_ production. Crucially, a moderately negative CB potential (close to 0.69 V vs. NHE, pH = 0) facilitates this selective 2e^−^ process while avoiding over-reduction. In contrast, the indirect ORR pathway involves a side-on adsorption configuration, which enables symmetrical interaction with both O atoms, favoring sequential 1e^−^ reductions first to •O_2_^−^, then •OOH, and finally to H_2_O_2_. This process requires a more negative CB to drive each electron transfer and stabilizes the radical intermediates through strong surface adsorption. However, excessively strong O_2_ adsorption or overly negative CB positions may promote a competing four-electron (4e^−^) reduction, leading to H_2_O instead of H_2_O_2_.

**Figure 4:**
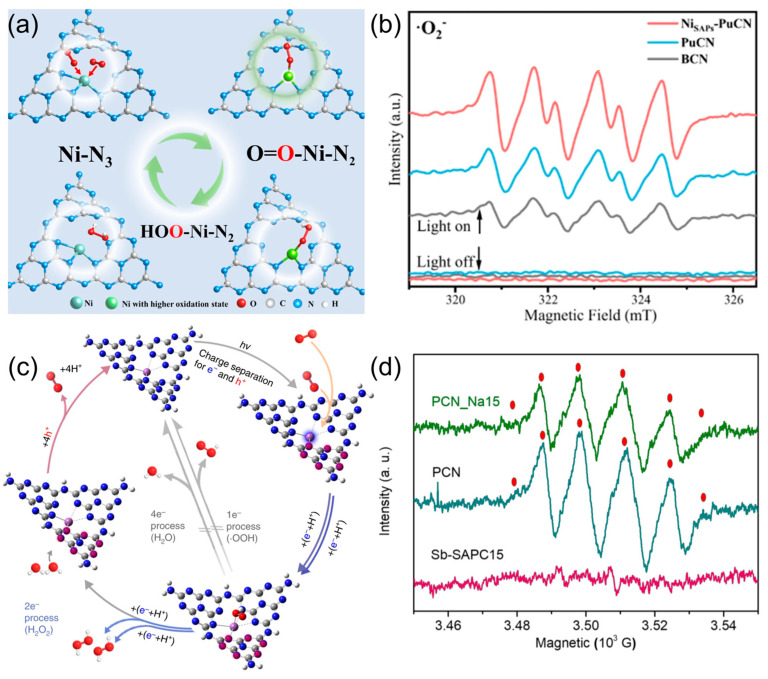
The indirect 2e^−^ ORR pathways for production of H_2_O_2_: (**a**) schematic diagram of single-atom Ni structure evolution of Ni_SAPs_-PuCN, (**b**) EPR signals of •O_2_^−^ of Ni_SAPs_-PuCN in the presence of DMPO. Reproduced from ref. [81] with permission from Springer Nature. The direct 2e^−^ ORR pathways for production of H_2_O_2_: (**c**) schematic diagram of single-atom Sb structure evolution of Sb-SAPC, (**d**) EPR signals of •O_2_^−^ of Sb-SAPC in the presence of DMPO. Reproduced from ref. [80] with permission from Springer Nature.

As illustrated in Figure 4a,b, Ni single atoms embedded in Ni_SAPs_-PuCN serve as electron reservoirs that facilitate the sequential single-electron reduction of O_2_. EPR spectroscopy under light irradiation reveals a strong signal for •O_2_^−^, indicating that •O_2_^−^ is indeed the dominant intermediate in this system [81]. The enhanced •O_2_^−^ signal, together with the band alignment and adsorption mode, strongly supports the indirect ORR route, where Ni centers modulate local charge density to stabilize radical intermediates. In contrast, as shown in Figure 4c,d, Sb single-atom Sb-SAPC exhibits no detectable EPR signal for •O_2_^−^, indicating that the photocatalytic reaction bypasses radical intermediates entirely. The introduction of Sb atoms optimizes O_2_ adsorption into an end-on configuration with moderate interaction strength, which promotes a concerted 2e^−^ reduction to H_2_O_2_ [80]. The authors demonstrate that Sb-SAPC can be synthesized at scale using a bottom-up wet chemical method with NaSbF_6_ and melamine, successfully producing 100 g in a single batch. Advanced characterization techniques confirm that over 99.6% of Sb atoms are atomically dispersed, ensuring high uniformity. The catalyst exhibits excellent stability, retaining over 95% activity after multiple photocatalytic cycles, and shows consistent performance across different batches. These findings validate the reproducibility and durability of Sb-SAPC, positioning it as a scalable and reliable single-atom catalyst for sustainable H_2_O_2_ production. This system exemplifies the direct ORR pathway, where appropriate orbital overlap and frontier energy matching between the Sb site and O_2_ molecule enable efficient and selective H_2_O_2_ generation. In addition to the typical oxygen reduction routes, photocatalytic H_2_O_2_ generation can also proceed through oxidative pathways involving the direct oxidation of water molecules (pathway III) or hydroxide ions (pathway IV). These pathways are thermodynamically uphill and typically overlooked in conventional ORR systems.

### 3.3. Regulation Toward Selective ^1^O_2_ Formation

^1^O_2_ is the first excited state of molecular oxygen and exhibits unique electrophilic reactivity and a long diffusion length, making it a crucial ROS for applications in pollutant degradation, antimicrobial therapy, organic synthesis, and photodynamic cancer therapy [9,10,11,12,13,46,49,82,83,84,85,86,87,88,89,90]. Compared to other ROS such as •OH and •O_2_^−^, ^1^O_2_ is non-radical, highly selective in oxidation reactions, and more stable under physiological and environmental conditions. Therefore, selectively regulating its formation in photocatalytic systems is essential for targeting oxidative processes that avoid excessive mineralization or indiscriminate damage.

**Figure 5:**
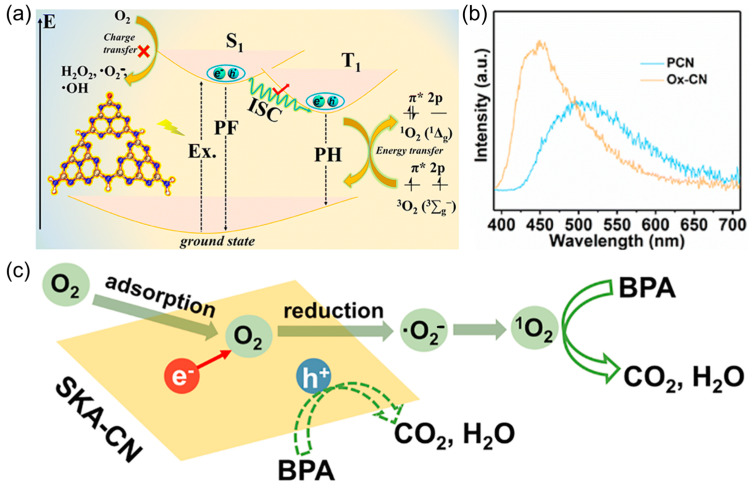
(**a**) The generation mechanism of ^1^O_2_ via the direct energy transfer pathway on Ox-CN. (**b**) The phosphorescence spectra of PCN and Ox-CN. Reproduced from ref. [46] with permission from Royal Society of Chemistry. (**c**) The generation mechanism of ^1^O_2_ via indirect electron-hole pathway via •O_2_^−^ oxidation on SKA-CN for degradation of BPA. Reproduced from ref. [86] with permission from Elsevier.

Figure 5a illustrates the direct energy transfer pathway for ^1^O_2_ generation on carbonyl-functionalized carbon nitride (Ox-CN) [46]. In this system, molecular oxygen is activated not by charge transfer, but via an energy transfer process. Upon photoexcitation, Ox-CN generates excitons, which undergo intersystem crossing (ISC) from singlet (S) to triplet (T) states. The carbonyl groups introduced into the PCN backbone serve as spin-orbit coupling (SOC) centers, significantly enhancing ISC efficiency by reducing the S-T energy gap. The triplet excitons transfer energy to the triplet ground state of molecular oxygen, promoting it to generating ^1^O_2_. This direct sensitization mechanism is further corroborated by the phosphorescence spectra shown in Figure 5b. Compared to pristine PCN, Ox-CN exhibits a stronger phosphorescence signal, which is a signature of an enhanced triplet-state population. Such a strategy not only enables efficient ^1^O_2_ production under visible light but also avoids the generation of potentially harmful radicals like •OH or •O_2_^−^, thereby improving the selectivity and safety of photocatalytic processes. Figure 5c illustrates an alternative and equally compelling pathway for ^1^O_2_ formation [86]: the indirect hole-driven oxidation of •O_2_^−^. In this mechanism, photogenerated electrons in the CB of chemically modified PCN first reduce molecular oxygen to •O_2_^−^. Subsequently, this superoxide radical anion is oxidized by VB holes to yield ^1^O_2_, completing a two-step redox loop. The strategic promotion of •O_2_^−^ generation and its subsequent oxidation by photogenerated holes offers a redox-closed-loop mechanism that maximizes charge carrier utilization while minimizing energy waste and radical overproduction. This indirect route not only expands the functional capacity of PCN but also provides a selectivity advantage in treating complex aqueous matrices, in which indiscriminate oxidants may generate harmful intermediates.

### 3.4. Regulation Toward Selective •OH Formation

Among all reactive oxygen species, the •OH stands out as one of the most potent oxidants, with a redox potential high enough to non-selectively oxidize almost any organic molecule. This unparalleled reactivity underpins its pivotal role in pollutant mineralization, advanced oxidation processes, and pathogen inactivation. However, the indiscriminate generation of •OH often compromises selectivity and efficiency, underscoring the need for the precise regulation of ROS pathways. Engineering PCN-based systems to selectively photogenerated charge carriers toward •OH production represents a transformative approach. By modulating surface chemistry, defect states, and interfacial dynamics, researchers can unlock new regimes of reactivity, in which •OH formation is not merely a consequence of photoredox activity but a finely orchestrated outcome of molecular design.

**Figure 6:**
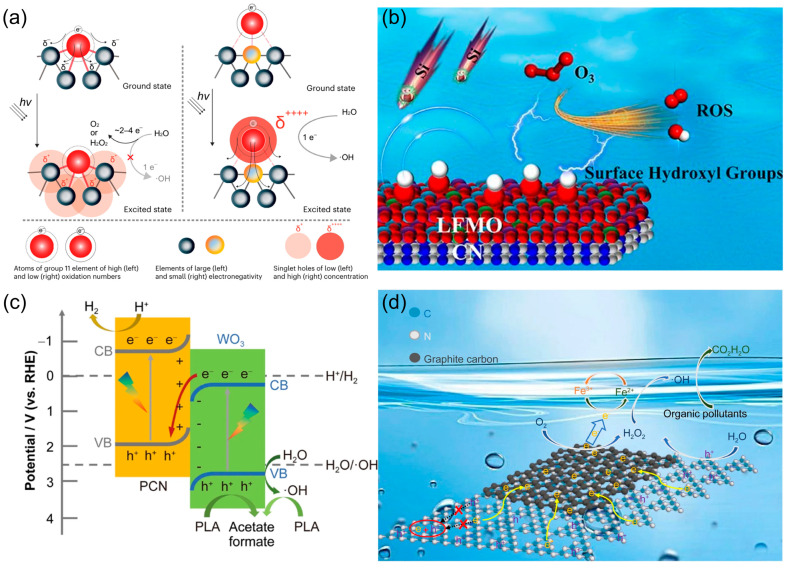
(**a**) The mechanism diagram of atomically dispersed low-valent Au boosts photocatalytic hydroxyl radical production. Reproduced from ref. [5] with permission from Springer Nature. (**b**) Surface hydroxylation promotes the conversion of ozone into hydroxyl radicals. Reproduced from ref. [91] with permission from Elsevier. (**c**) Constructing Z-type heterojunction to promote hydroxyl radical generation. Reproduced from ref. [92] with permission from Springer Nature. (**d**) Self-Fenton reaction promotes the generation of hydroxyl radicals for pollutant degradation. Reproduced from ref. [93] with permission from Springer Nature.

As illustrated in Figure 6, four complementary design strategies for enhancing photocatalytic •OH generation, each grounded in a distinct mechanistic pathway, are unified by the overarching principle of the precise regulation of charge carrier dynamics and interfacial chemistry. In the first, Teng et al. [5] engineered potassium-incorporated poly(heptazine imide) hosting atomically dispersed low-valent Au, whose K-N bonding environment stabilized Au^0^ species that trapped highly localized photogenerated holes; these sites drove an exceptionally efficient one-electron water oxidation reaction (WOR) to •OH (Figure 6a). As shown in Figure 6b, Wang et al. [91] coupled PCN to LaFe_0.26_Mn_0.74_O_3-δ_ perovskite to create LFMO-CN, where interfacial modulation increased surface -OH coverage; DFT revealed these hydroxyls lower the energy barrier for the O_3_ → HO_3_• → •OH pathway, enabling complete m-cresol removal, 70.2% mineralization, and a 2.4-fold improvement in silicon salt resistance across 15 reuse cycles. The band alignment enabled vectorial charge migration, as shown in Figure 6c. Wang et al. [92] fabricated a direct Z-scheme heterostructure between PCN and WO_3_. This configuration retained the strong oxidation potential of PCN’s valence band, while facilitating efficient electron transfer to the WO_3_ conduction band. As a result, the system achieved a 3.5-fold increase in PLA photoreforming activity under visible light. Mechanistic studies identified h^+^ and •OH as the dominant oxidative species, driving both hydrogen generation and the production of value-added formate/acetate. Chen et al. [93] enhanced a self-Fenton PCN platform by introducing a graphitic carbon interlayer (CUCN-2%). This modification improved photogenerated electron transfer to Fe^3+^, thereby accelerating the Fe^3+^/Fe^2+^ redox cycle (Figure 6d). The faster cycle promoted in situ H_2_O_2_ activation, yielding abundant •OH radicals as the primary oxidative species. As a result, the system achieved higher H_2_O_2_ utilization efficiency and RhB mineralization levels (63.77% in 3 h) triple those of pristine PCN.

Thus, while enhancing •OH formation via water oxidation ensures a sustainable electron source and avoids the accumulation of intermediates, it often suffers from sluggish kinetics and high overpotentials, whereas promoting •OH production through H_2_O_2_ decomposition offers higher efficiency under mild conditions but sacrifices atom economy and may generate competing pathways.

## 4. Applications of ROS from PCN

**Figure 7:**
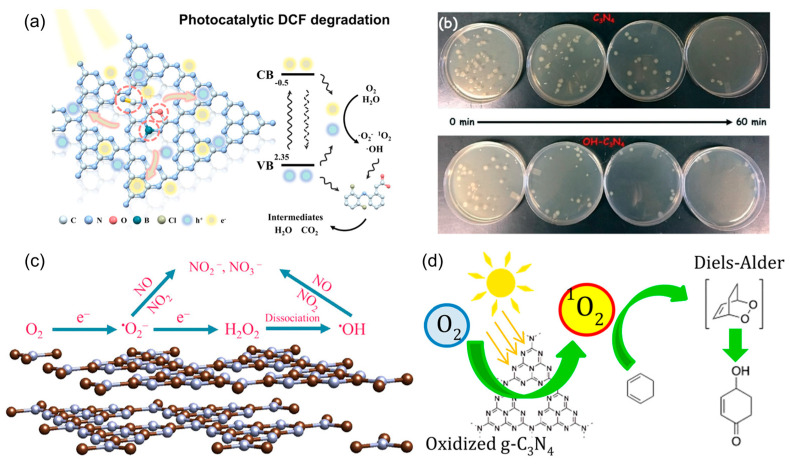
Applications of ROS from PCN. (**a**) Diclofenac degradation. Reproduced from ref. [67] with permission from Elsevier. (**b**) Photocatalytic disinfection. Reproduced from ref. [94] with permission from Royal Society of Chemistry. (**c**) Photocatalytic NO removal. Reproduced from ref. [95] with permission from Elsevier. (**d**) The organic synthesis for D-A reaction. Reproduced from ref. [96] with permission from ACS Publications.

Reactive oxygen species serve as powerful agents driving diverse photocatalytic applications. Harnessing their unique redox potentials, lifetimes, and selectivities enables tailored pathways for targeted outcomes. By tuning photocatalyst composition and structure to favor a specific ROS, PCN-based systems can move beyond conventional pollutant mineralization. This capability enables the selective oxidation of fine chemicals and the late-stage functionalization of complex molecules. In addition, such systems can drive integrated energy–environment processes, including simultaneous hydrogen production and organic waste valorization. Mapping the distinct reactivity modes of each ROS, ranging from precise single-electron transfer to broad-spectrum radical attack, provides a strategic toolkit for aligning each species with its optimal transformation, thereby expanding the technological reach of photocatalytic platforms. By aligning the intrinsic reactivity of each ROS with the chemical demands of a given target, whether requiring selective single-electron oxidation, controlled two-electron pathways, or indiscriminate radical attacks, PCN systems can be engineered for high selectivity, efficiency, and functional breadth. Such strategic matching maximizes the utility of PCN in pollutant removal, disinfection, and synthesis, while expanding its technological reach into domains traditionally inaccessible to conventional photocatalysts (Figure 7).

In water treatment, defect engineering, non-metal doping, and crystallinity optimization were integrated into a triple-strategy PCN design that simultaneously enhanced charge separation and oxygen activation capacity, enabling rapid diclofenac degradation while co-producing H_2_O_2_ as a valuable byproduct, shown in Figure 7a [67]. Its high mineralization efficiency, coupled with excellent reusability and stability, demonstrates the potential of tailored defect–dopant–crystallinity engineering as a general blueprint for environmental remediation and sustainable oxidant generation. In microbial disinfection, the surface hydroxyl functionalization of PCN increased hydrophilicity and introduced additional electron-rich sites, facilitating efficient •OH formation under visible light, shown in Figure 7b [94]. This structural tuning shortened the diffusion distance between generated ROS and bacterial membranes, resulting in accelerated inactivation kinetics and improved efficacy against both Gram-negative and Gram-positive strains. For air purification, precise control over ROS evolution and transformation enabled efficient photocatalytic NO removal [95]. As shown in Figure 7c, it revealed that •O_2_^−^ initiates NO oxidation to NO_2_, which then undergoes further oxidation by •OH to yield nitrate species, thereby minimizing harmful NO_2_ accumulation. This sequential ROS cascade provides a mechanistic blueprint for designing photocatalysts that avoid secondary pollution. Beyond environmental remediation, PCN has been utilized as a visible-light photosensitizer for organic synthesis, particularly in generating ^1^O_2_ under mild and metal-free conditions. This approach enabled Diels–Alder cycloadditions with a broad substrate scope and high chemo-selectivity, highlighting the capacity of ^1^O_2_ to promote pericyclic reactions without harsh reagents or elevated temperatures (Figure 7d) [96]. Together, these examples demonstrate that by tuning the generation, conversion, and release of specific ROS, PCN-based photocatalysts can serve as versatile, high-performance platforms that bridge environmental technology and synthetic chemistry.

Reactive oxygen species (ROS) have emerged as pivotal agents in addressing critical challenges across energy and chemical manufacturing sectors. In pollutant degradation, ROS facilitate the photocatalytic oxidation of alcohols to aldehydes, offering a sustainable alternative to traditional methods [97]. In hydrogen production, ROS are integral to photocatalytic systems that convert water into hydrogen gas under visible light, presenting a renewable energy solution [98]. Collectively, these applications underscore the transformative potential of ROS in advancing sustainable technologies across various industrial domains [99].

Stability and durability are central considerations for the practical deployment of PCN-based ROS photocatalysts. While impressive catalytic performances have been reported, the long-term reusability of PCN remains a challenge, as ROS can also attack the photocatalyst itself. A study by Li et al. [100] reported that hydroxyl radicals, formed under photocatalytic conditions, trigger the structural degradation of g-C_3_N_4_ via bond cleavage and ring opening, leading to diminished activity over repeated cycles. This highlights how powerful oxidants like •OH can erode the triazine network, undermining long-term performance, especially under photo-Fenton or high-flux radical environments. These changes decrease conjugation, lower carrier mobility, and shift band edges, leading to a progressive decline in ROS productivity and selectivity.

Single-atom catalysts (SACs) have attracted significant attention due to their high atomic efficiency and tunable electronic properties. However, their practical application is often limited by stability issues, particularly under harsh reaction conditions. One major challenge is metal leaching, as isolated metal atoms can detach from the support in acidic or oxidative environments, reducing catalytic activity and raising environmental concerns. Another concern is structural degradation, including the sintering or aggregation of single atoms, which diminishes the number of active sites, especially under high temperatures or reactive intermediates. Engineering the coordination environment of the metal sites has been demonstrated to stabilize the structure and preserve atomic dispersion.

## 5. Conclusions and Outlook

In this review, we have systematically examined the generation mechanisms and regulation strategies of various ROS on polymeric PCN-based photocatalysts. Beginning with a detailed analysis of individual ROS types, including •O_2_^−^, ^1^O_2_, H_2_O_2_, and •OH, we highlighted their distinct formation pathways and associated electronic and surface factors that govern their selectivity. Subsequently, we discussed targeted strategies for manipulating these ROS, such as conduction band engineering, surface site design, heterojunction construction, and defect modulation, emphasizing how these approaches enable precise control over ROS production. Finally, the diverse applications of PCN-derived ROS were surveyed, spanning environmental remediation, disinfection, air purification, and organic synthesis, demonstrating the versatile utility of PCN platforms when combined with selective ROS management.

ROS are pivotal intermediates in photocatalytic processes, enabling a wide range of applications from environmental remediation to chemical synthesis. PCN, with its unique metal-free framework and tunable electronic properties, has emerged as a versatile platform for selective ROS generation. Through sophisticated structural modulation, including defect engineering, heteroatom doping, and heterojunction construction, researchers have substantially enhanced the ability of PCN to produce distinct ROS with tailored reactivity profiles. These advances have opened new avenues for designing photocatalytic systems that achieve both high efficiency and specificity.

Recent advances in computational methods have opened new avenues for the rational design of PCN photocatalysts with tailored ROS generation profiles. Machine learning algorithms, trained on high-throughput experimental and computational datasets, can identify correlations between structural features, such as layer thickness, defect density, doping type, and surface functionalization, and ROS selectivity. This predictive capability allows researchers to prioritize synthetic targets with optimized electronic structures and interfacial properties, reducing the reliance on trial-and-error experimentation. Moreover, AI-driven models can simulate dynamic charge carrier behavior and predict the relative yields of ROS under varying light and environmental conditions. Integrating ML/AI with density functional theory (DFT) calculations or molecular dynamics further enables mechanistic insights into ROS formation pathways, including energy transfer processes and electron–hole coupling. Collectively, these data-driven strategies hold the potential to accelerate the discovery of next-generation PCN-based photocatalysts, achieving highly selective, efficient, and application-specific ROS generation while minimizing experimental effort.

Nonetheless, challenges remain in precisely controlling the generation and transformation of ROS to optimize selectivity and catalytic performance. The coexistence of multiple ROS pathways often complicates mechanistic understanding and can lead to undesired side reactions. Limitations inherent to PCN, such as moderate light absorption and charge transport, further restrict its photocatalytic potential. Addressing these challenges requires a multidisciplinary approach. Integrating advanced in situ characterization techniques with computational modeling will be essential to unravel the complex dynamics of ROS formation under realistic reaction conditions. At the same time, atomic-level manipulation of PCN’s structure, including the introduction of single-atom active sites and controlled defect distributions, will provide finer control over ROS pathways. Moreover, combining PCN photocatalysis with external stimuli such as applied bias, photothermal effects, or mechanical forces may unlock new regimes of ROS selectivity and efficiency. Importantly, future research must align material design with application-specific requirements, ensuring not only catalytic performance but also long-term stability, reusability, and environmental compatibility.

## Figures and Tables

**Table 1:** H_2_O_2_ generation pathways.

Pathway	Overall Reaction	Pathway Type	Feasibility on PCN	Selectivity
I. Stepwise reduction via •O_2_^−^/HO_2_·	O_2_ + e^−^ → •O_2_^−^ •O_2_^−^ + H^+^ → HO_2_· HO_2_· + e^−^ + H^+^ → H_2_O_2_	Radical-mediated, stepwise	High (common under aerobic conditions)	Moderate (possible ROS side reactions)
II. Direct 2e^−^ O_2_ reduction	O_2_ + 2H^+^ + 2e^−^ → H_2_O_2_	Non-radical, concerted 2e^−^ transfer	Moderate to High (requires PCET facilitation)	High (less radical leakage)
III. Water oxidation	2 H_2_O + 2 h^+^ → H_2_O_2_ + 2 H^+^	Oxidative, uphill reaction	Low (requires oxidation co-catalysts)	Low (competing O_2_ evolution)
IV. •OH coupling	H_2_O + h^+^ → •OH + H^+^ •OH + •OH → H_2_O_2_	Radical coupling, oxidative	Low (requires high •OH concentration)	Low (non-selective, degradation-prone)

## Data Availability

No new data were created or analyzed in this study.
